# CD44v9 Induces Stem Cell-Like Phenotypes in Human Cholangiocarcinoma

**DOI:** 10.3389/fcell.2020.00417

**Published:** 2020-06-03

**Authors:** Nattawan Suwannakul, Ning Ma, Kaoru Midorikawa, Shinji Oikawa, Hatasu Kobayashi, Feng He, Shosuke Kawanishi, Mariko Murata

**Affiliations:** ^1^Department of Environmental and Molecular Medicine, Mie University Graduate School of Medicine, Tsu, Japan; ^2^Graduate School of Health Science, Suzuka University of Medical Science, Suzuka, Japan; ^3^Faculty of Pharmaceutical Sciences, Suzuka University of Medical Science, Suzuka, Japan

**Keywords:** cholangiocarcinoma, CD44 variant 9, epithelial-mesenchymal transition, Wnt/β-catenin, cancer stem cell

## Abstract

**Background:** Our previous study demonstrated an overexpression of CD44 variant 9 (CD44v9) in human cholangiocarcinoma (CCA) tissues that was associated with inflammation-related tumor development. However, the participation of CD44v9 in cholangiocarcinogenesis remains poorly understood. Therefore, in this study, we examined the potential roles of CD44v9 in CCA cells to understand the carcinogenic mechanism.

**Methods:** Using normal cholangiocytes (MMNK1) and CCA cells (KKU213), the expression levels of CD44v9 and its related molecules were quantified through RT-qPCR and immunofluorescence (IF) staining. To evaluate its biological functions, we performed CD44v9 (exon 13) silencing using siRNA transfection, and assessed cell proliferation through MTT assay, cell migration and invasion by transwell technique, and carried out cell cycle analysis by flow cytometry. *In vivo* tumor growth was assessed by nude mouse xenografts, and histological and molecular changes were determined.

**Results:** KKU213 exhibited higher protein expression levels of CD44v9 than those of MMNK1 through IF staining. RT-qPCR analysis revealed that the mRNA expression level of CD44v9 was predominantly elevated in CCA cells along with its neighboring exons such as variant 8 and 10, minimally affecting the standard form of CD44. CD44v9 silencing could regulate redox system in CCA cells by reducing the expression levels of SOD3 and cysteine transporter xCT. CD44v9 silencing suppressed the CCA cell proliferation by induction of apoptosis and cell cycle arrest. Migration and invasion were decreased in CD44v9 siRNA-treated CCA cells. CD44v9 downregulation inhibited CCA tumor growth in mouse xenografts. IF analysis demonstrated the histological changes in xenograft tissues such as an increase in connective tissues through collagen deposition and reduction of hyaluronic acid synthesis through CD44v9 silencing. CD44v9 knockdown *in vitro* and *in vivo* increased E-cadherin and reduced vimentin expression levels, resulting in reduction of epithelial-mesenchymal transition (EMT) process. Moreover, CD44v9 modulated Wnt10a and β-catenin in tumorigenesis.

**Conclusion:** Our results indicate that CD44v9 plays a potential role in CCA development by the regulation of cell proliferation and redox balancing. CD44v9 silencing may suppress tumor growth, migration and invasion through EMT: a finding that could potentially be applied in the development of targeted cancer therapy.

## Introduction

Cancer stem cell (CSC), a subpopulation of tumor cells, is associated with biological processes of carcinogenesis by facilitating self-renewal, tumor metastasis, tumor recurrence, and drug resistance (Phi et al., 2018). CD44 is abundantly expressed in normal tissues. It has been recognized as a CSC marker in various types of cancers, and found to play a role in various biological processes, such as cancer proliferation and metastasis. The variant isoforms of CD44 (CD44v) generated by alternative splicing have been reported to reveal higher aggressive potential in tumor as compared to the standard isoform (CD44s) (Prochazka et al., 2014). Among ten CD44 variants, CD44v9 has been closely associated with cellular processes and tumorigenicity comprising cell proliferation, metastasis, and tumor invasiveness through epithelial-mesenchymal transition (EMT) (Yasui et al., 1998; Miwa et al., 2017; Vaquero et al., 2017; Taniguchi et al., 2018). Kiuchi et al. (2015), have found that the expression of CD44v9 was upregulated during the mitotic phase of pancreatic cancer cells. Furthermore, CD44v9-positive cells were shown to possess the ability to suppress the production of reactive oxygen species (ROS), resulting in the therapeutic resistance, recurrence, and metastasis of tumors (Wada et al., 2013; Ogihara et al., 2019). In our previous study, CD44v9 was overexpressed in *Opisthorchis viverrini*-related cholangiocarcinoma (OV-CCA) tissues and was proposed to be a potential CSC marker for OV-CCA (Suwannakul et al., 2018). Investigation of the correlation between CD44v9 and CCA could prove meaningful in the determination and development of appropriate cancer therapy for CCA. However, the biological role of CD44v9 in CCA development still remains unclear.

We hypothesize that the downregulation of CD44v9 may be involved in suppression of CCA development. Therefore, we investigated the phenotypic changes by introducing small interfering RNA (siRNA) into CCA cells. The results showed that a downregulation of CD44v9 led to a suppression of cell proliferation along with an enhancement of cell apoptosis and cell cycle arrest. CD44v9 siRNA also inhibited cell migration and invasion. Furthermore, CD44v9 silencing in nude mouse xenograft model resulted in the suppression of tumor growth and histological changes in the tumor stroma. In order to address one of the biological alterations in CD44v9-related CCA development, we found the possible link between EMT phenotype and Wnt signaling pathway for tumorigenic regulation.

## Materials and Methods

### Cell Culture

Two different cell lines were used in this study, including human cholangiocyte cell line (MMNK1) and cholangiocarcinoma cell line (KKU213) that were obtained from the Japanese Collection of Research Bioresources Cell Bank (Osaka, Japan). The cell lines were maintained in DMEM (Thermo Fisher Scientific, Waltham, MA, United States) supplemented with 100 U/mL penicillin, 100 mg/mL streptomycin, and 5% fetal bovine serum (FBS) for MMNK1 or 10% for KKU213 cells (MP Biomedicals, Santa Ana, CA, United States) in a humidified incubator at 37°C and 5% CO_2_.

### siRNA Synthesis and Transfection

For CD44v9 silencing, exon 13 siRNAs were designed and synthesized (MISSION siRNA, Sigma-Aldrich, MO, United States), and the sequences used were as follows: siRNA#1; (sense) 5′-CUUCUACUCUGACAUCAAG-3′, (antisense) 5′-CUUGAUGUCAGAGUAGAAG-3′. siRNA#2; (sense) 5′-GAGCUUCUCUACAUCACAU-3′, (antisense) 5′-AUGUGAUGUAGAGAAGCUC-3′. The MMNK1 and KKU213 cells were seeded in a six-well plate at a density of 0.5 × 10^6^ cells/well using the antibiotics-free medium supplemented with FBS and incubated overnight at 37 °C. Subsequently, the cells were treated with either the negative control (*SIC*-001-10, Merck, Darmstadt, Germany) or the target siRNA (10 μM, a final concentration is 10 nM) using lipofectamine 3000 reagent (Invitrogen, Waltham, MA, United States) following the manufacturer’s instructions. After 24 h post-transfection, cells were harvested and prepared for the subsequent experiments.

### Immunofluorescence (IF) Staining

The treated cells with siRNA and the non-treated cells were seeded in an 8 chamber glass slide at 0.2 × 10^6^ cells/mL and incubated at 37°C overnight. The cells were then fixed with 4% formaldehyde in PBS for 10 min, and treated with 1% skim milk for 1 h. Following this, the cells were incubated with primary antibodies (Supplementary Table S1A) overnight. The secondary antibodies (Supplementary Table S1A) were subsequently added to the cells, followed by incubation for 3 h in dark. The slides were then mounted with DAPI-fluoromount-G (Southern Biotech, Birmingham, AL, United States) and the stained cells were observed under fluorescent microscope (Olympus, Tokyo, Japan). The quantitative analysis of fluorescent intensity was performed using ImageJ and a relative ratio of intensity was calculated in comparison to that of the nuclear staining of DAPI, since Zhang et al. (2019), indicated that there was no difference between DAPI and GAPDH, as a reference for the adjustment of cell number.

### RNA Extraction and Quantitative Real-Time PCR

The specific Taqman probe and primers were obtained from Applied Biosystems (Waltham, MA, United States), including CD44 [exon 3-4 (Hs01075864_m1), exon 12-13 (Hs010- 75856_m1), exon 13-14 (Hs-b01075857_m1), exon 14-15 (Hs01075858_m1)], CAT (Hs00156308_m1), GPx1 (Hs00- 829989_gH), SOD1 (Hs00533490_m1), SOD2 (Hs00- 167309_m1), SOD3 (Hs00162090_m1), xCT (Hs00921938_m1), and GAPDH (Hs99999905_m1).

Total RNA was extracted from MMNK1 and KKU213 cells using TRIZOL reagent (Thermo Fisher Scientific) and integrity was confirmed using the 1% agarose gel electrophoresis. cDNA was synthesized from the isolated RNA (2 μg) using the High-Capacity RNA-to-cDNA Kit (Applied Biosystems), and 1 μL of cDNA was used for each PCR reaction using TaqMan Universal Master Mix II (Applied Biosystems). The conditions used for real-time PCR were as follows: 95°C for 10 min followed by two-step PCR (95°C for 15 s and 60°C for 1 min) for 50 cycles using StepOnePlus Real Time PCR System (Applied Biosystems). In each experiment, negative control was used where nuclease free water was added in the reaction mixture instead of the template. The samples were run in duplicates and the levels of expression were normalized with GAPDH.

### Cell Proliferation

One day before conducting the experiment, 1.5 × 10^3^ cells/well of siRNA-transfected MMNK1 and KKU213 cells were plated in 96-well plate for 24 h. Subsequently, the experimental medium (antibiotics-free medium supplemented with FBS) was replaced by complete medium (medium supplemented with FBS and antibiotics) and the cells were cultured for 24, 48, 72, and 96 h. At each time-point of post-transfection, 10 μL of 5 mg/mL MTT solution (Merck) was added and the cells were incubated at 37 °C for 4 h. Subsequently, the solution was removed, and 150 μL DMSO was added and the absorbance was measured at a wavelength of 570 nm using the microplate reader (Bio-Rad, Hercules, CA, United States). The experiment was performed in six replicates using independent conditions.

### Assessment of Cell Apoptosis and Cell Cycle Using Flow Cytometry

Early apoptotic and late apoptotic cells were identified using Muse Annexin V & Dead Cell Kit (MCH100105, MUSE, Merck, Waltham, MA, United States). KKU213 cells were transfected with siRNA for 24 h, the experimental medium was replaced by complete medium and incubated for 72 h at 37°C and 5% CO_2_ in an incubator. Treated cells were harvested and resuspended in a serum-containing medium. Cell solution was mixed with Muse^TM^ Annexin V & Dead Cell Reagent, incubated for 20 min at room temperature in dark, and analysis was done using Muse Cell Analyzer (Luminex, Austin, TX, United States). For analysis of apoptotic cells, debris were excluded by gating.

The percentage of cells at each stage was analyzed using MUSE Cell Cycle Kit (MCH100106, MUSE, Merck). Briefly, the siRNA treated cells were harvested and washed with PBS. Then cells were resuspended in 70% cold ethanol and incubated overnight at -20°C. Before analysis, the ethanol-fixed cells were washed with PBS and resuspended in Muse^TM^ Cell Cycle Reagent following the manufacturer’s instructions. Subsequently, the cells were incubated in dark at room temperature for 30 min, cell cycle analysis was performed using MUSE Cell Analyzer, and the data were evaluated using ModFit LT 3.0 software (Verity Software House, ME, United States). All experiments were performed in triplicates. For analysis of cell cycle, debris and dead cells were excluded by gating.

### Cell Migration and Invasion

The migration and invasion assays for KKU213 cells were performed in triplicates using a 24-well transwell chamber, 8 μm pore size (Corning Incorporated, Corning, NY, United States). The interior of inserts and wells of Corning BioCoat Matrigel Invasion Chambers were rehydrated with serum-free medium for 2 h at 37°C and 5% CO_2_. The medium was removed and 750 μL of complete medium containing 10% serum was added into each well. Subsequently, 4 × 10^4^ cells/500 μL of siRNA treated cells in serum-free medium were plated on control insert (for migration) or Matrigel insert (invasion). After an incubation of 24 h, non-migratory or non-invaded cells were gently removed from inserts with a cotton swab. The migratory or invaded cells were fixed and stained by cell stain solution (Cell Biolabs, San Diego, CA, United States) for 10 min at room temperature and the inserts were allowed to air dry. The number of migratory or invaded cells were counted under microscope using 5 microscopic fields/sample. The percentage of invasion was calculated as (cell number of invasion)/(cell number of migration) × 100 to avoid the effect of a decrease in cell viability by CD44v9 siRNA.

### Western Blot

Total protein of cells was extracted by RIPA buffer (Cell Signaling Technology, Dancers, MA, United States) containing protease inhibitor (100 mM phenylmethylsulfonyl fluoride). For the fraction of nuclear and cytosolic protein, NucBuster Protein Extraction Kit was used (Novagen, Darmstadt, Germany), according to the instruction. Briefly, the packed cells were resuspended in 150 μL NucBuster Reagent 1, incubated on ice for 5 min and centrifuged at 12,000 rpm, 4°C for 5 min. The supernatant (cytoplasmic fraction) was separated. The pellet was resuspended in 77 μL mixture of Protease Inhibitor Cocktail, DTT and NucBuster Extraction Reagent 2, incubated on ice for 5 min and centrifuged at 12,000 rpm, 4°C for 5 min. The supernatant (nuclear extract) was transferred and prepared for the assay. The protein concentration was evaluated by Coomassie Protein Assay Kit (ThermoFisher Scientific, MA, United States) and 20–30 μg of total protein in 4X SDS sample buffer (ThermoFisher Scientific) was loaded onto 5–20% polyacrylamide gel (Wako, Osaka, Japan). Electrophoresis was performed at 600 V, 40 mA for 35 min. Proteins were transferred to PVDF membranes (Merck Millipore, MA, United States) at 600 V, 100 mA for 35 min. The membranes were then blocked with 5% skim milk in Tris-buffered saline (TBS) containing 0.1% Tween 20 (TBS-T) for 30 min at room temperature, and incubated with primary antidodies (Supplementary Table S1B) at 4°C overnight. The membranes were washed with TBS-T and incubated with horseradish peroxidase-conjugated secondary antibodies (Supplementary Table S1B) for 1 h at room temperature. After washing with TBS-T, the signals were visualized by enhanced chemiluminescence reagent (GE Healthcare, CA, United States) and detected by LAS4000mini (Fujifilm, Tokyo, Japan). The fold change of intensity was evaluated using ImageJ and normalized to GAPDH, and Histone H3 was used for nuclear protein.

### Drug Resistance

The transfected cells were seeded in 96-well plate at 2000 cells/well. Cells were treated with various concentrations of fluorouracil (5-FU) (Wako) at 37°C, 5% CO_2_ for 24 h. Cell viability was evaluated by adding 10 μL of 5 mg/mL MTT solution (Merck Millipore) and incubated at 37°C for 4 h. After removed cell solution and added DMSO, the reaction was measured at a wavelength of 570 nm.

### Spheroid Formation Assay

Spheroids were established in agarose-based 96-well plates. Briefly, 80 μL of the sterile 1% agarose (Lonza, Gampel, Switzerland) (w/v in PBS) was added into 96-well plate and incubated at 37°C for 2 h. The transfected cells were harvested by trypsinization and washed with PBS. One hundred microliters of cell suspension (2 × 10^4^ cells/well) were seeded in 96-well plate containing agarose gel. The microplate was centrifuged at 1000 rpm for 5 min. Clusters of cells were observed after 72 h of seeding under a phase contrast inverted microscope (Olympus). The diameter of tumor spheroid was recorded and determined using ImageJ.

### Cysteine Assay

The quantification of cysteine was determined using Cysteine assay kit (Abcam, Cambridge, United Kingdom). Cells were treated with siRNA condition before experiment. Cells were harvested and lysed by resuspension in assay buffer and homogenized on ice. Supernatant was collected and deproteinized using 10 kDa spin column (Merck Millipore). Then, the eluent was treated following the instruction, and then measured at Ex/Em = 365/450 nm in kinetic mode at room temperature. The concentration of cysteine in sample was calculated from standard curve.

### Tumor Xenograft Model Analysis

Four-week-old male athymic BALB/c nude mice (*n* = 10 mice per condition) were purchased from Japan SLC Inc. (Hamamatsu, Japan). All protocols for animal studies were approved by the committee of animal center of Mie University, Mie, Japan (Approval no. 26-19-sai2-hen1). The mice were maintained under specific pathogen-free conditions. Each mouse was subcutaneously injected with 2 × 10^6^ cells in the flank region. KKU213 cells treated with negative control siRNA was inoculated at the right flank and KKU213 cells treated with CD44v9 siRNA#1 was inoculated at the left flank. The body weight and tumor growth were monitored every 2 days. Tumor volume was measured using a caliper and calculated by the following formula: volume (mm^3^) = 0.5 × length × width^2^. After 2 weeks, all mice were sacrificed and the tumor tissues were collected and weighed. Each tumor was divided into two parts for IF staining and for mRNA expression analysis.

### Histological and Immunohistochemical Studies

Mouse xenograft tumors were fixed with 4% formaldehyde in PBS for 1 day. Following dehydration and paraffin infiltration, tumors were embedded in paraffin blocks and were then sectioned to 5 μm thickness using Leica Microsystems (Wetzlar, Germany). Histopathological appearance of mouse tumors was evaluated by hematoxylin & eosin (H&E) staining, immunofluorescence (IF), and trichrome staining methods.

For IF, the paraffin embedded mouse tumor sections were deparaffinized in xylene and series of alcohol. After the retrieval of heat-induced epitopes using microwave at 500W for 5 min and blocking with 1% skim milk in PBS pH 7.4, sections were incubated overnight with primary antibodies (Supplementary Table S1A) followed by secondary antibodies (Supplementary Table S1A) for 2 h. Nuclei were stained with DAPI and tissues were observed under fluorescent microscope (Olympus). The quantitative analysis of fluorescent intensity was performed using ImageJ and a relative ratio of intensity was calculated in comparison to that of the nuclear staining of DAPI, as a reference for the adjustment of cell number (Zhang et al., 2019).

The collagen fibers were determined using Trichrome Stain Kit (Modified Masson’s; ScyTek Laboratories, Logan, UT, United States) following manufacturer’s instructions. Each tissue sample of ten tumors per condition was observed under microscope using 20X objective magnification at least different five areas. The percentage of collagen positive area (blue staining) was quantified from 10 tumors per group using ImageJ.

### Statistical Analysis

Data are presented as the mean ± standard error of the mean (SEM) from at least three independent experiments. Statistical analysis was performed using an SPSS software version 23.0 (IBM Corporation, United States). Comparisons of data between groups were analyzed using Student’s *t*-test. A paired *t*-test was used to compare the differences between the tumor size and volume in the negative control siRNA and CD44v9-siRNA treated cells in a mouse. A *p*-value of less than 0.05 was considered to be statistically significant.

## Results

### CD44 Isoforms and Antioxidant System Expression Profiles Distinguish Between Normal Cholangiocytes and Cholangiocarcinoma Cells

**FIGURE 1:**
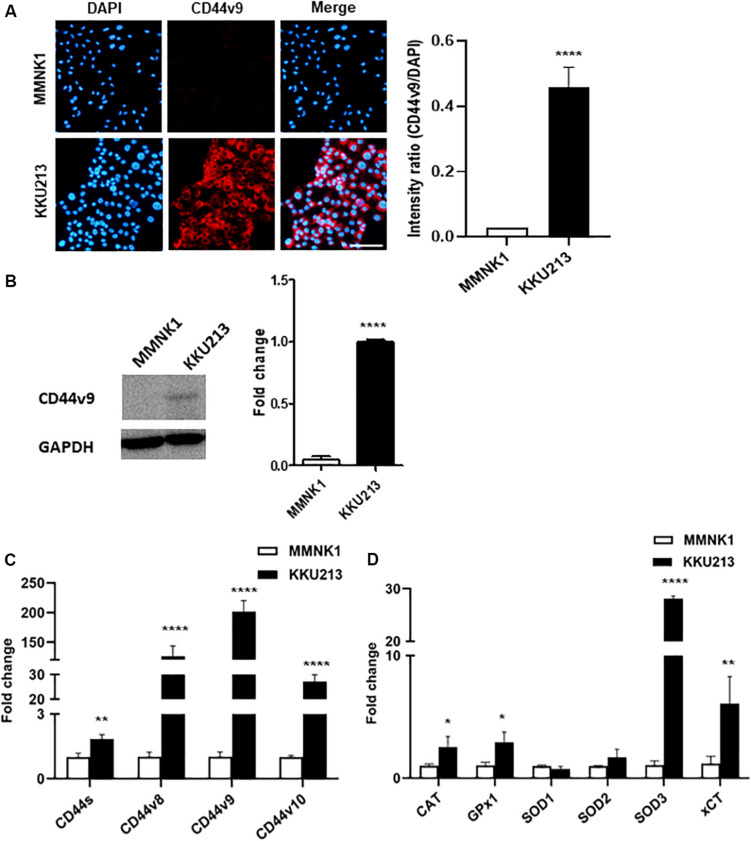
Expression of CD44 isoforms and antioxidant-related genes in normal bile duct (MMNK1) cells and CCA (KKU213) cells. **(A)** Protein level of CD44v9 (scale bars; 50 μm) and quantitative intensity was evaluated as a relative ratio (CD44v9/DAPI). **(B)** Blot images of CD44v9. **(C)** mRNA levels of standard and variant isoforms of CD44 gene. **(D)** mRNA levels of antioxidant system. The values are the mean ± SEM. *p < 0.05, **p < 0.01, ****p < 0.0001 vs. MMNK1 cells.

In the present study, CD44v9 protein expression in normal cholangiocytes (MMNK1) and CCA cells (KKU213) was analyzed by IF staining. An overexpression of CD44v9 was observed in KKU213 cells with a significant increase in fluorescent intensity as compared to MMNK1 cells. Consistent with IF results (Figure 1A), western blot analysis (Figure 1B) demonstrated that CD44v9 was higher level in KKU213 cells than in MMNK1 cells.

The mRNA expression analysis of various CD44 isoforms was carried out by RT-qPCR containing standard form (primers targeting exon 3–4) and variant 9 (exon 13–14) with neighboring exons of variant 8 (exon 12–13) and 10 (exon 14–15). Predominantly higher expression of variant 9 (201.3 ± 18.8 folds) was observed in KKU213 cells with higher expression levels of variant 8 (126.1 ± 17.4-fold), and variant 10 (27.2 ± 2.8-fold), in comparison to the normal bile duct cells (Figure 1C). Standard form of CD44 was slightly higher in KKU213 cells (1.8 ± 0.2-fold) as compared to MMNK1 cells.

RT-qPCR for antioxidant enzyme genes (Figure 1D) showed higher expression levels of CAT, GPx1, and SOD3 in KKU213 cells (2.5 ± 0.8, 2.9 ± 0.8 and 28.1 ± 0.5-fold, respectively), while there was no significant difference in SOD1 (0.8 ± 0.2 folds) and SOD2 (1.7 ± 0.6-fold). Likewise, CCA cells showed a high expression of xCT (6.1 ± 2.2-fold) that is involved in intracellular redox homeostasis.

### Expression Profiles of CD44 Isoforms and Antioxidant System Are Altered in CD44v9 Silencing Cells

**FIGURE 2:**
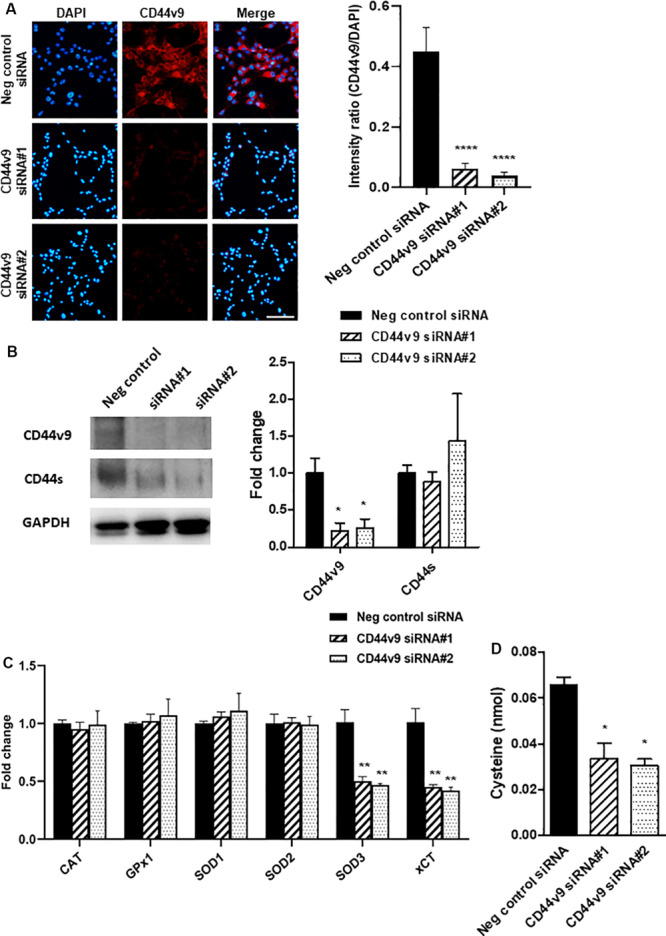
Expression changes in CCA cells with CD44v9 knockdown condition. **(A)** Protein level of CD44v9 analyzed through IF staining (scale bars; 50 μm) and quantitative intensity was evaluated as a relative ratio (CD44v9/DAPI). **(B)** Blot images of CD44v9 and CD44s. Although the blots only depict one experiment, CD44v9 consistently decreased by CD44v9 siRNA. The expression of CD44s varied among the experimental replicates, so on average of the three experiments, there was no clear difference. **(C)** mRNA levels of antioxidant system. **(D)** Quantification of cysteine. The values are the mean ± SEM. *p < 0.05, **p < 0.01, ****p < 0.0001 vs. negative control siRNA cells.

The efficiency of CD44v9 knockdown in CCA cells was verified by analyzing the level of protein expression by immunostaining. CD44v9 staining was observed in negative control siRNA-treated KKU213 cells, while a significant lower fluorescent intensity was observed in CD44v9 siRNA-treated KKU213 cells (Figure 2A). Western blot analysis revealed the lower protein level of CD44v9 in CD44v9 silencing cells similar to IF staining, but interestingly, CD44s protein levels were similar between CD44v9 and negative control siRNA treatment (Figure 2B).

Comparison between negative control siRNA and CD44v9 siRNA in KKU213 cells showed that the expression levels of CAT, GPx1, SOD1, and SOD2 were not altered. Interestingly, CD44v9 knockdown in KKU213 cells reduced the expression levels of SOD3 and xCT (Figure 2C). These results implied that CD44v9 downregulation influenced the CD44v9 expression directly, and also regulated the cellular antioxidant system in CCA cells.

Furthermore, the evaluation of cysteine was performed using fluorometric assay. The amount of cysteine was reduced in CD44v9 knockdown cells compared to the negative control (Figure 2D). These findings suggest that CD44v9 elevates xCT expression with higher cysteine levels in cancer cells which may be associated with antioxidative properties.

### CD44v9 Downregulation Inhibits Cell Proliferation

**FIGURE 3:**
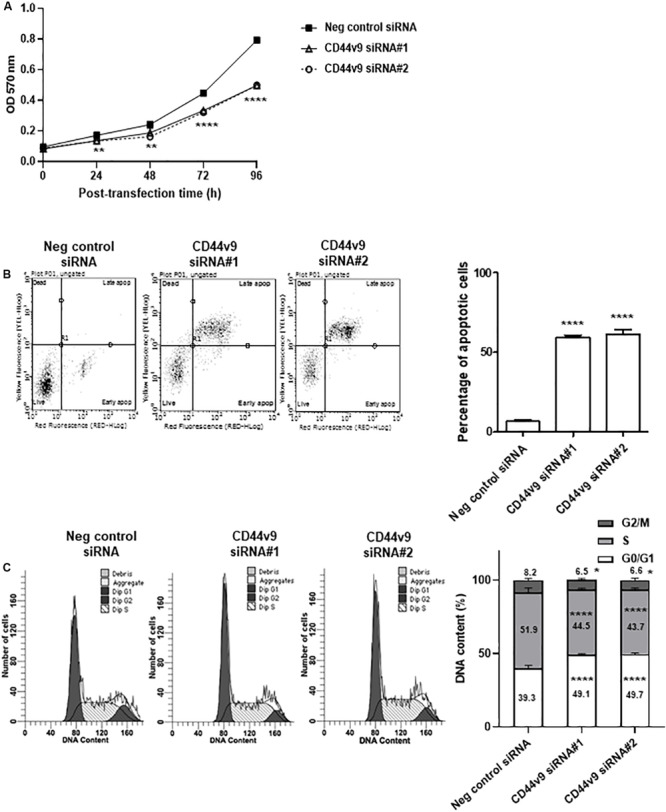
Cell proliferation study of CD44v9 knockdown condition in CCA cells. **(A)** KKU213 cells were treated with siRNA, and cell growth was evaluated through MTT assay. **(B)** Diagram and percentage of cell apoptosis. Data show four cell populations of viable cells (lower left quadrant), early apoptotic cells (lower right quadrant), necrotic cells (upper left quadrant), and late apoptotic cells (upper right quadrant). *Y*-axis is labeled for viability (dead cell marker; 7-AAD) and *X*-axis is labeled for cell apoptosis (phosphatidylserine-binding protein; Annexin V). Bar chart presents the percentage of total apoptotic cells (early and late apoptotic cells). **(C)** Diagram and percentage of cell cycle. Histogram shows the number of cells (*Y*-axis) and DNA content (*X*-axis) with G0/G1 phase (tallest peak), G2/M phase (rightmost peak) and S phase (between G0/G1 and G2/M phases). The values are the mean ± SEM. *p < 0.05, **p < 0.01, ****p < 0.0001 vs. negative control siRNA cells.

To identify cell proliferation in CD44v9 knockdown condition, the cell viability rate was evaluated using MTT spectrophotometric assay. Viability in CD44v9-knockdown KKU213 cells was lower than that of negative control cells (Figure 3A). In contrast, CD44v9-knockdown did not affect the viability of MMNK1 cells (Supplementary Figure S1). These results indicate that the overexpression of CD44v9 may be related to the proliferation of CCA cells, suggesting that CD44v9 can be a cancer-specific therapeutic target.

The flow cytometry analysis was performed to validate the effect of CD44v9 downregulation on cell death and cell cycle regulation. Consequently, a significant increase in apoptotic cells was observed in CD44v9 knockdown cells by siRNA#1; 59.0 ± 2.1 and siRNA#2; 62.0 ± 4.2%, while low levels were observed in negative control cells (6.9 ± 1.9%) (Figure 3B). These results indicated that CD44v9 knockdown could promote cell death in CCA cells.

The effect of CD44v9 downregulation on cell cycle showed an increased population of G0/G1 (siRNA#1; 49.1 ± 0.9 and siRNA#2; 49.7 ± 0.7%) as compared to the negative control siRNA cells (39.9 ± 2.1%). Furthermore, the proportions of S and G2/M phases were decreased in CD44v9 siRNA-treated cells (S phase as siRNA#1; 44.5 ± 0.9%, siRNA#2; 43.7 ± 1.3% and G2/M phase as siRNA#1; 6.5 ± 1.1%, siRNA#2; 6.6 ± 1.2%) as compared to the negative control siRNA cells (S phase as 51.9 ± 2.9% and G2/M phase as 8.2 ± 1.1%) (Figure 3C). These data suggest that downregulation of CD44v9 could induce CCA cell cycle arrest at the G0/G1 phase.

### CD44v9 Knockdown Suppresses Cancer Cell Migration and Invasion

**FIGURE 4:**
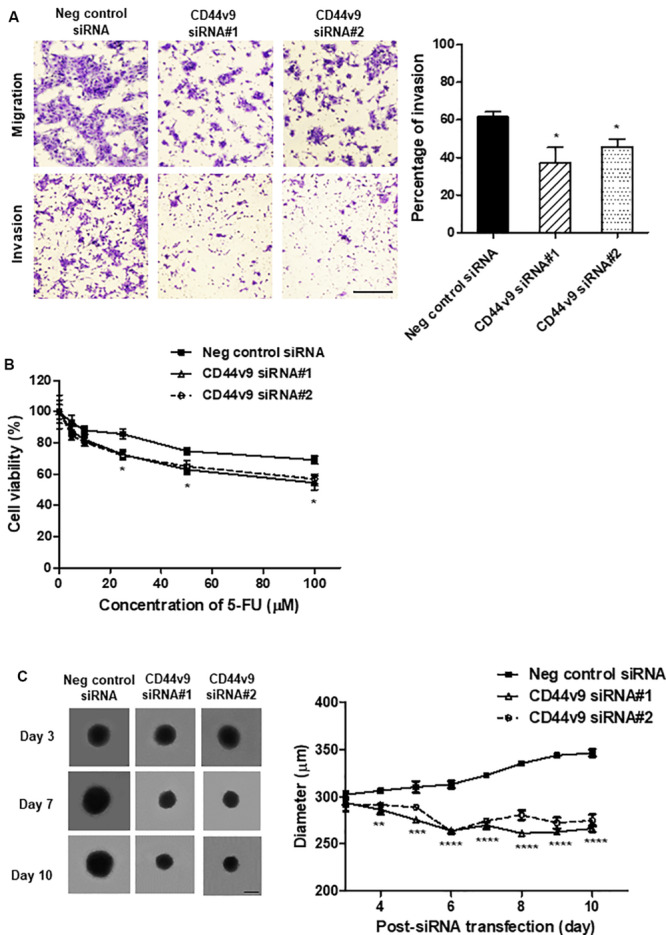
Study of cell migration/invasion and stem cell-like phenotypes in CD44v9 knockdown condition in CCA cells. **(A)** Morphology of stained migratory and invaded cells (scale bars; 50 μm), and the percentage of invasion. **(B)** Cell viability with various concentrations of 5-FU for 24 h. **(C)** Spheroid formation and quantitative diameter of spheroid cells. The values are the mean ± SEM. *p < 0.05, **p < 0.01, ***p < 0.001, ****p < 0.0001 vs. negative control siRNA cells.

To investigate the effects of CD44v9 silencing on CCA cells, the migration and invasion assays were performed using the Control and Matrigel Invasion Chambers, respectively. CD44v9 knockdown inhibited migratory and invasive abilities as compared to negative control siRNA treated cells (Figure 4A). The percentage of invasion significantly decreased in CD44v9 knockdown cells (siRNA#1; 37.15 ± 11.83% and siRNA#2; 45.60 ± 5.88%) as compared to the negative control siRNA treated cells (61.64 ± 3.82%) (Figure 4A, right).

### CD44v9 Knockdown Enhances Drug Sensitivity

siRNA transfected cells were treated with various concentrations of 5-FU and cell viability was determined 24 h later. 5-FU inhibited the proliferation of negative control cells with IC_50_ (the half maximal inhibitory concentration) value at 375.0 μM. In CD44v9 downregulated cells, the IC_50_ value was improved by decreasing to 133.7 and 175.9 μM in CD44v9 siRNA#1 and siRNA#2, respectively. These data imply that downregulation of CD44v9 may enhance the sensitivity of 5-FU against cancer development (Figure 4B).

### CD44v9 Knockdown Diminishes the Formation of Tumor Spheroid

The spheroid cells were generated in both of negative control and CD44v9 knockdown cells on day 3 after siRNA transfection. The spheroids of negative control cells were spontaneously grown by 14.62% (from 302.27 ± 6.01 μm to 346.47 ± 6.58 μm) on day 10. In contrast, the diameter of spheroids in CD44v9 silencing cells were decreased by 9.30 and 5.69% in siRNA#1 (from 293.59 ± 6.36 μm to 266.30 ± 5.99 μm) and siRNA#2 (from 292.15 ± 12.75 μm to 274.73 ± 10.90 μm), respectively (Figure 4C). These appearances indicate downregulation of CD44v9 could moderate tumor spheroid formation, one of stem cell-related characteristics.

### CD44v9 Knockdown Attenuates EMT and Wnt/β-Catenin Signaling in CCA Cells

**FIGURE 5:**
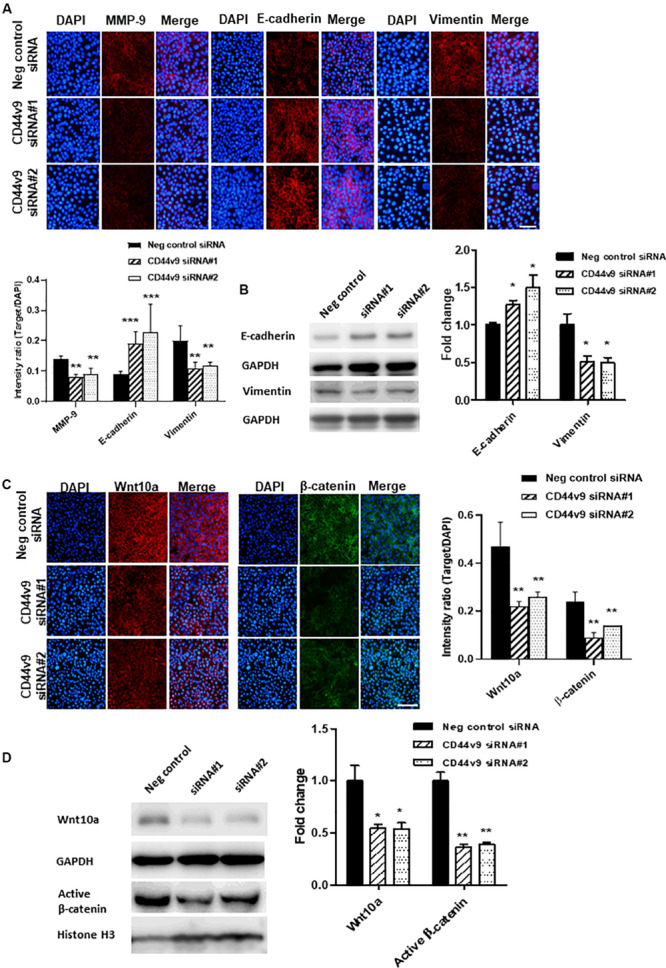
Effect of CD44v9 knockdown cells on EMT and Wnt signaling pathway. **(A)** IF staining with quantitative intensity and **(B)** blot images of MMP9, E-cadherin, vimentin. **(C)** IF staining (scale bars; 50 μm) with quantitative intensity and **(D)** blot images of Wnt10a and nuclear active β-catenin. The values are the mean ± SEM. *p < 0.05, **p < 0.01, and ***p < 0.001 vs. negative control siRNA cells.

Based on the prior results of cell migration and invasion, we presumed that CD44v9 could regulate CCA progression through EMT-associated cellular processes. To verify this hypothesis, the expression levels of matrix metalloproteinase (MMP)-9 and EMT-related genes were determined by IF staining and western blot. CD44v9 siRNA cells illustrated the reduced intensities of MMP-9 and vimentin, and a significantly elevated expression of E-cadherin (Figures 5A,B). Furthermore, we observed an altered Wnt/β-catenin signaling. We performed preliminarily RNA sequencing analysis (data not shown), and found that Wnt10a was inhibited in CD44v9 knockdown cells when compared to negative control. Thus, we focused on Wnt10a in our study for CD44v9-related Wnt/b-catenin signaling pathway. By IF and blot images, the diminution of Wnt10a and β-catenin (active β-catenin) were detected in CD44v9 knockdown cells as compared to the negative control siRNA cells (Figures 5C,D). These results indicate that the inhibitory effect of the CD44v9 downregulation on cancer properties may be involved in the EMT process and the Wnt/β-catenin signaling pathway.

### CD44v9 Knockdown Retards Tumor Growth in Mouse Xenograft

**FIGURE 6:**
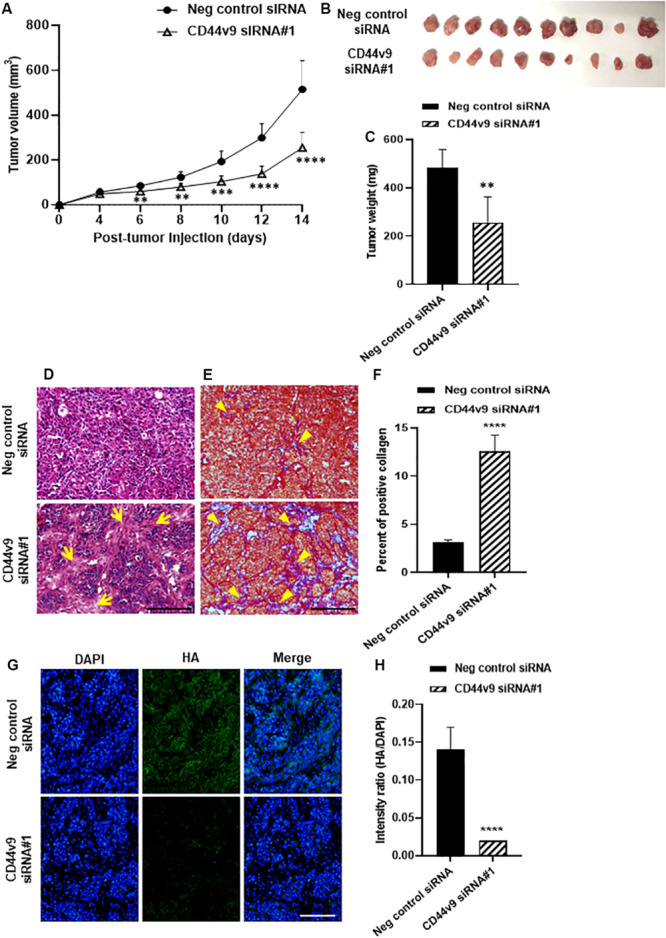
Tumor xenograft study of CD44v9 knockdown condition. **(A)** Tumor volume, **(B)** resected tumors, and **(C)** tumor weight. **(D)** Hematoxylin and eosin staining, yellow arrows indicate connective tissues. **(E)** Trichrome staining, yellow arrowheads indicate collagen fiber (blue staining) (scale bars; 50 μm). **(F)** Amount of collagen was quantified as blue intensity per tissue area using ImageJ. **(G)** Immunofluorescence staining of HA. **(H)** Quantitative intensity evaluated as a relative ratio (HA/DAPI). The values are the mean ± SEM. **p < 0.01, ***p < 0.001, ****p < 0.0001 vs. negative control siRNA group.

To evaluate growth inhibition *in vivo*, CD44v9 siRNA#1-treated cells were injected subcutaneously into the left flank of nude mice while negative control siRNA-treated cells were injected in the right flank of the same mice. During tumor growth, the individual bodyweight was not different among the animals (data not shown). In comparison to the negative control, the tumor growth rate was markedly decreased in CD44v9 knockdown group demonstrated by smaller tumor volume (Figure 6A) and tumor weight (control; 484.8 ± 75.0 vs. CD44v9 knockdown; 257.3 ± 105.5 mg) (Figures 6B,C). CD44v9 downregulation in the xenograft tissues was confirmed by IF staining (Supplementary Figure S2).

Histological study showed a high density of cancer cells in the negative control group and an increase in connective tissues (arrows) in CD44v9 knockdown group (Figure 6D). Trichrome staining revealed abundant collagen deposition (arrowheads, Figure 6E) and the percentage of collagen area was significantly increased in CD44v9 knockdown group (Figure 6F). Hyaluronic acid (HA) content was significantly decreased in CD44v9 knockdown group (Figures 6G,H). These results indicate that the downregulation of CD44v9 could suppress tumor progression in nude mouse xenograft model, by enhancing the collagen deposition and inhibiting the HA synthesis.

### CD44v9 Knockdown Suppresses EMT and Wnt/β-Catenin Signaling *in vivo*

**FIGURE 7:**
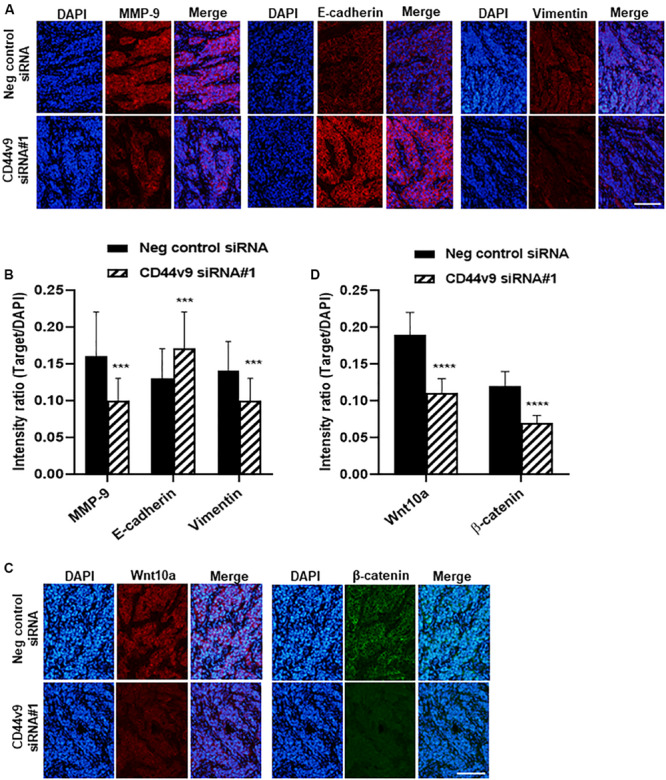
Immunofluorescence staining of relative molecules in EMT and Wnt signaling pathway. **(A)** Protein levels of MMP9, E-cadherin, vimentin and **(C)** Wnt10a and β-catenin (scale bars; 50 μm). **(B,D)** Quantitative intensity was evaluated as a relative ratio (target molecule/DAPI). The values are the mean ± SEM. ***p < 0.001, ****p < 0.0001 vs. negative control siRNA cells.

Since EMT signaling was observed to participate in CCA development during *in vitro* CD44v9 downregulation, mouse xenograft tumor tissues were used for the assessment of the expression levels of EMT molecules. In negative control treatment, an overexpression of MMP-9 was observed, and it was decreased in tissues of CD44v9 siRNA treatment group. Furthermore, CD44v9 knockdown increased E-cadherin expression and reduced vimentin expression as compared to the control (Figures 7A,B), resulting in reduction of the EMT process. These *in vivo* observations could support our assumption of the possible inhibitory mechanism of CD44v9 silencing through EMT regulation.

To better understand the mechanism of growth inhibition in CD44v9 downregulation, we investigated whether CD44v9 was able to regulate the activity of Wnt/β-catenin pathway in CCA. We validated the protein expression levels of Wnt10a and β-catenin in mouse xenograft tissues by performing a comparison between CD44v9 knockdown and control groups by IF staining. Reduced signal intensities of Wnt10a and β-catenin were observed in CD44v9 knockdown tissues as compared to the negative control tissues (Figures 7C,D). These results indicate that CD44v9 overexpression in CCA cells is considerably associated with the stem cell-like properties, possibly through the Wnt/β-catenin signaling pathway.

## Discussion

Our previous study had revealed that the overexpression of CD44v9 was associated with tumorigenesis and inflammation in CCA tissues, especially in OV-CCA patients (Suwannakul et al., 2018). In the present study, we focused on CD44v9 function in CCA development. Initially, we confirmed that CD44v9 was expressed considerably in CCA cells (KKU213) as compared to the normal bile duct cells (MMNK1), and siRNA transfection for exon 13 effectively decreased the expression levels of CD44v9 and neighboring isoforms. Silencing of CD44v9 inhibited cell proliferation by promoting apoptosis and cell cycle arrest. Furthermore, CD44v9 siRNA reduced the properties of the stem cell-like phenotypes including cell migration, invasion, drug resistance, and spheroid formation. These observations of *in vitro* CD44v9 knockdown correlated with the *in vivo* tumor growth and histological changes.

Development of specific gene silencing using siRNA-based strategy offers the posttranscriptional regulation of gene expression. As a cancer therapeutic approach, it has a potential to suppress the functional genes involved in cell proliferation, apoptosis, and drug resistance (Oh and Park, 2009; Guo et al., 2013). We analyzed these phenotypes and tumorigenic hallmarks of CCA cells through the knockdown of CD44v9, and our results exhibited a correlation that supports the inhibitory mechanism of *in vitro* and *in vivo* tumorigenicity models. Its effect related to tumor growth delay may be associated with the enhancement of cell cycle arrest and cell death in cancer cells. Interestingly, CD44v9 knockdown had no effect on normal bile duct cells, indicating that CD44v9 plays an essential role specifically in cancer cell proliferation. These results also indicate that CD44v9 silencing could be used as a therapeutic target for the treatment of CCA.

Antioxidant system is associated with stem cell-like properties in various types of cancer. In CSCs, ROS levels were significantly lower as a result of robust free radical scavenging system that protected the tumor cell DNA from endogenous or exogenous ROS and oxidative damage (Li et al., 2017), leading to resistance to chemoradiotherapy. In the present study, RT-qPCR analysis revealed an upregulation of CAT, GPx1, xCT, and SOD3 in CCA KKU213 cells as compared to the normal bile duct MMNK1 cells. Among them, CD44v9 silencing could suppress xCT and SOD3 expression levels, suggesting the direct association of the antioxidant system with CD44v9. The relative expression of CD44v9 has been associated with xCT system in gastric cancer and hepatocellular carcinoma (Miyoshi et al., 2018; Wada et al., 2018). As a result of this interaction, a combination of CD44v9 and xCT was found on the tumor cell surface. It suggests that the reduction of CD44v9 level could alter the xCT expression. SOD3 mainly exists in the extracellular space that binds to cell surface proteoglycans, and its overexpression has been implicated in liver cancer (Griess et al., 2017). However, SOD3 expression may be affected by CD44v9 knockdown through an unknown mechanism. Resistance to oxidative stress is a key mechanism for tumor treatment failure. Relevantly, an xCT inhibitor sulfasalazine suppressed the CCA cell growth (Thanee et al., 2016). CD44v9 siRNA-induced suppression of xCT and SOD3 expressions may lead to an increase in the ROS level, which could improve CCA treatment.

Among the stem cell-like properties, the interaction of CD44 receptor with its ligands such as HA, collagens, and MMPs contributes significantly in the processes of migration and invasion in cancers (Senbanjo and Chellaiah, 2017). A major ligand HA can bind to CD44 resulting in conformational change and activation of various signaling pathways involved in cell motility and adhesion. Auvinen and colleagues have found a negative or slight HA expression in normal ductal epithelium of mammary glands, whereas elevated HA level was observed in malignant breast tumors associated with cancer metastasis (Auvinen et al., 2000). Moreover, in bladder cancer, the downregulation of CD44 variant caused due to a decrease in HA production had a regulatory role in cell growth and apoptosis (Golshani et al., 2008). In addition, collagen degradation has a crucial role in inhibiting the tumor migration and invasion. Human breast cancer cells (MDA-MB-231 cells) that have a high CD44 expression possess a potential activity of MMPs that facilitates invasiveness and CD44 depletion attenuated cell invasion through the reduction of collagen degrading enzymes (Montgomery et al., 2012). Moreover, Evanko et al. (2015), reported the inhibition of HA synthesis by promoting fibronectin and collagen deposition. Avnet and Cortini reviewed that the pericellular matrix formed by HA and its multivalent interactions with tumor receptors not only intensify malignancy and therapy resistance, but also exacerbate the stem cell-like phenotype by increasing stem-cell marker genes and promoting invasion and migration, via EMT (Avnet and Cortini, 2016). These reports could support our results in case of CD44v9 knockdown inhibited CCA invasion and migration through HA suppression and collagen replacement.

EMT, a cellular phenotypic conversion by which epithelial cells transform into highly motile and invasive mesenchymal cells, has been implicated in inducing the stem cell-like phenotypes in cancer cells by facilitating the initiation of tumor invasion and metastasis development (Lamouille et al., 2014; Xue et al., 2015). Major hallmarks of EMT include the loss of epithelial cell junction and polarity, increase of cell motility and enhanced cellular MMPs activity (Lu et al., 2018). Based on the phenotypic changes in cells and tissues, the present study illustrated that high CD44v9 condition stimulates the EMT process by diminishing epithelial marker, E-cadherin, and elevating the mesenchymal marker, vimentin, with a simultaneous increase in MMP-9 expression. CD44v9 silencing conversely altered all makers, and reduced CCA cell migration and invasion, indicating a close correlation of EMT and CD44v9.

Intriguingly, several signaling pathways have been involved in the regulation of EMT and acquiring the CSC properties, such as Wnt/β-catenin, TGFβ, Hedgehog and Notch (Wang and Zhou, 2013; Wang et al., 2015; Barat et al., 2017). Zhang K et al. reported that the activation of Wnt ligands promoted the ability of self-renewal in prostate CSCs (Zhang et al., 2017). In nasopharyngeal carcinoma, the silenced β-catenin suppressed the stem cell-like abilities of cell proliferation and migration (Jiang et al., 2016). Moreover, several studies reported a positive correlation between β-catenin and CD44 due to the upregulation of β-catenin and CD44 resulting in an increased proliferation *in vitro* (Wei et al., 2019) and *in vivo* (Sarkar et al., 2011). Interestingly, the overexpression of Wnt10a could stimulate migration and proliferation of transformed esophageal cells, and induce a population of self-renewal CD44^High^ cells (Long et al., 2015). In the present study, we found that both Wnt10a and β-catenin were downregulated in CD44v9 silenced cells *in vitro* and *in vivo*. CD44v9 may induce Wnt signaling, resulting in the nuclear translocation of β-catenin which further activates cellular processes including cell proliferation, migration and invasion in CCA through EMT activation.

## Conclusion

**FIGURE 8:**
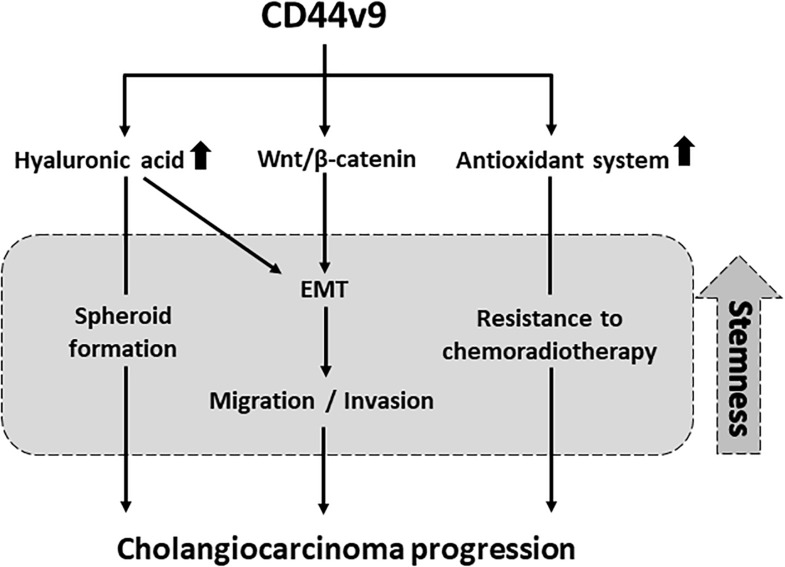
Proposed pathway in which CD44v9 induces stem cell-like phenotypes.

In conclusion, CD44v9 expression plays a potent role in the induction of stem cell-like phenotypes to be enhancing the CCA progression, including cell proliferation, migration, invasion, and redox balancing through interaction with ligand along with EMT (Figure 8). We propose that the inhibition of targeted CD44v9 could effectively be applied in development of treatment strategies for CCA, and could provide a fundamental basis for the development of therapeutic agents.

## Data Availability Statement

The datasets generated for this study are available on request to the corresponding author.

## Ethics Statement

The *in vivo* study was approved by the Committee of Animal Center of Mie University (approval no. 26-19-sai2-hen1).

## Author Contributions

MM, KM, and NS were involved in the design of the study, interpretation of data, and revision of manuscript. NS prepared the manuscript, performed the experiments, and data analysis. NM helped conduct the immunofluorescence staining and participated in animal tissue preparation. FH helped performing western blot analysis. SO, HK, and SK assisted in manuscript editing. All authors read and approved the final manuscript.

## Conflict of Interest

The authors declare that the research was conducted in the absence of any commercial or financial relationships that could be construed as a potential conflict of interest.

## Supplementary Material

FIGURE S1 Cell proliferation study of CD44v9 knockdown condition in normal bile duct cell (MMNK1).

FIGURE S2 Immunofluorescence staining of CD44v9 in mouse xenograft tissues. The values are the mean ± SEM. ^∗∗^*p* < 0.01 vs. negative control siRNA cells.

TABLE S1 List of primary and secondary antibodies used for **(A)** immunofluorescence staining and **(B)** western blot analysis.
