# Supplementary figures and images for: CD44v9 Induces Stem Cell-Like Phenotypes in Human Cholangiocarcinoma
Source: Front Cell Dev Biol. 2020 Jun 3;8:417. doi: 10.3389/fcell.2020.00417 (PMC7283556; doi:10.3389/fcell.2020.00417)

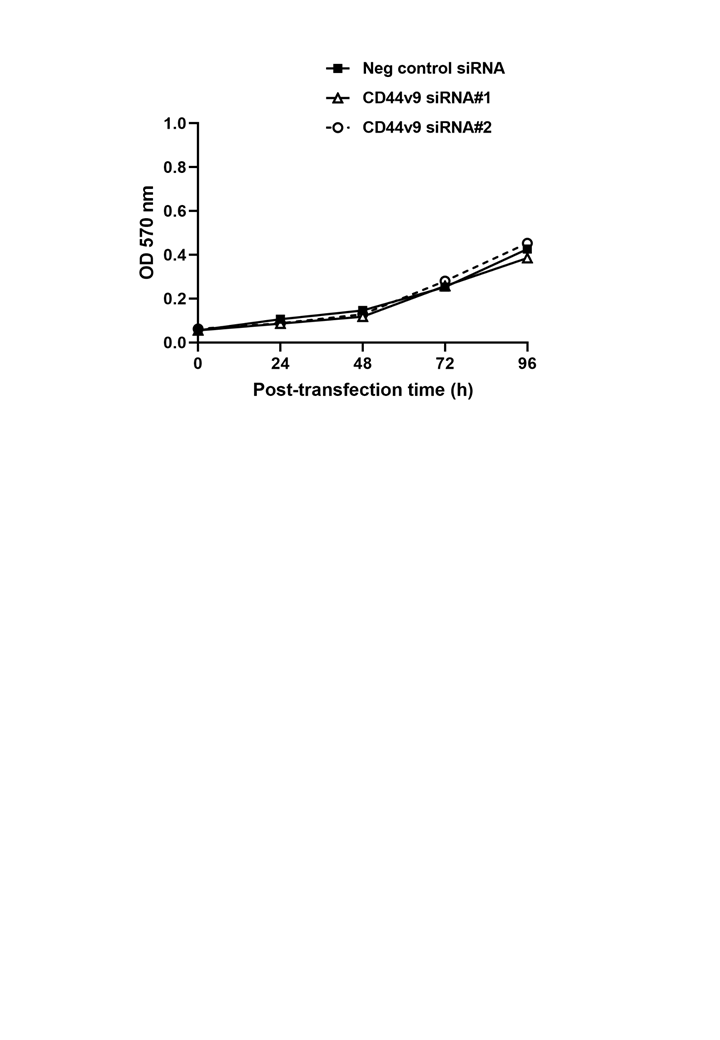

Supplement: FIGURE S1 — Cell proliferation study of CD44v9 knockdown condition in normal bile duct cell (MMNK1). [file Image_1.TIF]

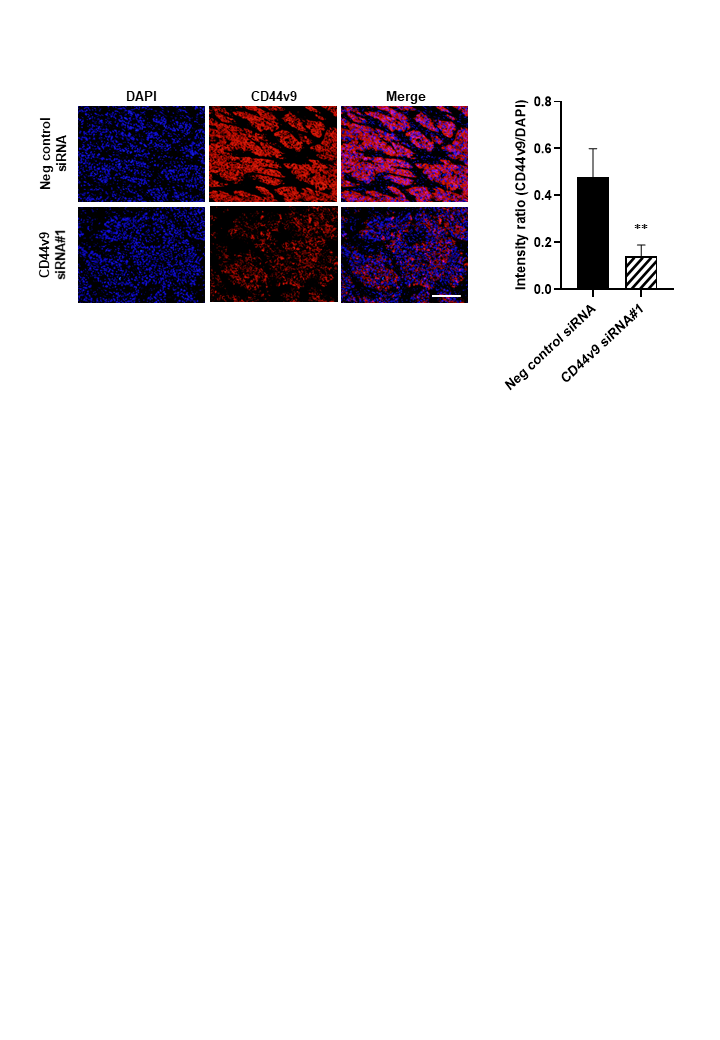

Supplement: FIGURE S2 — Immunofluorescence staining of CD44v9 in mouse xenograft tissues. The values are the mean ± SEM. ∗∗p < 0.01 vs. negative control siRNA cells. [file Image_2.TIF]
