# Supplementary material for: CD44v9 Induces Stem Cell-Like Phenotypes in Human Cholangiocarcinoma
Source: Front Cell Dev Biol. 2020 Jun 3;8:417. doi: 10.3389/fcell.2020.00417 (PMC7283556; doi:10.3389/fcell.2020.00417)
Supplement: TABLE S1 — List of primary and secondary antibodies used for (A) immunofluorescence staining and (B) western blot analysis. [file Data_Sheet_1.PDF]

**Table S1.**

A) List of primary and secondary antibodies used for immunofluorescence staining.

| **Primary antibody** | **Host species** | **Antibody dilution** | **Manufacturer** | **Catalog no.** |
|---|---|---|---|---|
| CD44v9 | Rat | 1:200 | Cosmo Bio | LKG-M001 |
| Hyaluronic acid | Sheep | 1:300 | Abcam | Ab53842 |
| MMP-9 | Goat | 1:50 | Santa Cruz | SC-6840 |
| E-cadherin | Rabbit | 1:100 | Cell Signaling Technology | 3195 |
| Vimentin | Rabbit | 1:100 | Cell Signaling Technology | 5741 |
| Wnt10a | Rabbit | 1:100 | Abcam | Ab106522 |
| β-catenin | Mouse | 1:200 | BD Biosciences | 610154 |
| **Secondary antibody** | | **Antibody dilution** | **Manufacturer** | **Catalog no.** |
| donkey anti-rat Alexa 594 | | 1:400 | Invitrogen | A21209 |
| donkey anti-sheep Alexa 488 | | 1:400 | Abcam | Ab150177 |
| goat anti-rabbit Alexa 594 | | 1:400 | Invitrogen | A11012 |
| goat anti-mouse Alexa 488 | | 1:400 | Invitrogen | A11001 |

B) List of primary and secondary antibodies used for Western blot analysis.

| **Primary antibody** | **Host species** | **Antibody dilution** | **Manufacturer** | **Catalog no.** |
|---|---|---|---|---|
| CD44v9 | Rat | 1:500 | Cosmo Bio | LKG-M001 |
| CD44s | Mouse | 1:100 | R&D Systems | BBA10 |
| E-cadherin | Rabbit | 1:1000 | Cell Signaling Technology | 3195 |
| Vimentin | Rabbit | 1:500 | Cell Signaling Technology | 5741 |
| Wnt10a | Rabbit | 1:1000 | Abcam | Ab106522 |
| Active β-catenin | Mouse | 1:1000 | Merck Millipore | 05-665 |
| GAPDH | Rabbit | 1:1000 | Abcam | Ab9485 |
| Histone H3 | Rabbit | 1:1000 | Cell Signaling Technology | 4499 |
| **Secondary antibody** | | **Antibody dilution** | **Manufacturer** | **Catalog no.** |
| goat anti-rat | | 1:2000 | Cell Signaling Technology | 7077 |
| donkey anti-rabbit | | 1:10000 | Santa Cruz | sc-2313 |
| goat anti-mouse | | 1:10000 | Santa Cruz | sc-2031 |
